# Therapeutic Effects and Biomarkers in Sublingual Immunotherapy: A Review

**DOI:** 10.1155/2012/381737

**Published:** 2012-03-05

**Authors:** Takashi Fujimura, Yoshitaka Okamoto, Masaru Taniguchi

**Affiliations:** ^1^Research Center for Allergy and Immunology, Yokohama Institute, RIKEN, 1-7-22 Suehiro-cho, Tsurumi-ku, Yokohama, Kanagawa 230-0045, Japan; ^2^Department of Otolaryngology, Head and Neck Surgery, Graduate School of Medicine, Chiba University, 1-8-1 Inohara, Chuo-ku, Chiba 260-8670, Japan

## Abstract

Immunotherapy is considered to be the only curative treatment for allergic diseases such as pollinosis, perennial rhinitis, asthma, and food allergy. The sublingual route is widely applied for immunotherapy for allergy, instead of the conventional administration by subcutaneous route. A recent meta-analysis of sublingual immunotherapy (SLIT) has shown that this approach is safe, has positive clinical effects, and provides prolonged therapeutic effects after discontinuation of treatment. However, the mechanism of SLIT and associated biomarkers are not fully understood. Biomarkers that change after or during SLIT have been reported and may be useful for response monitoring or as prognostic indicators for SLIT. In this review, we focus on the safety, therapeutic effects, including prolonged effects after treatment, and new methods of SLIT. We also discuss response monitoring and prognostic biomarkers for SLIT. Finally, we discuss immunological mechanisms of SLIT with a focus on oral dendritic cells and facilitated antigen presentation.

## 1. Introduction

Allergic rhinitis is the most prevalent type I allergy, and pollen grains, mite, and mold are common causative allergens for seasonal or perennial rhinitis. Antihistamines, leukotriene inhibitors, and nasal steroids are commonly used to treat respiratory allergy, but these drugs sometimes have side effects that induce impaired performance [1, 2]. Almost 100 years have passed since the first report of immunotherapy for pollinosis in 1911 [3]. Subsequently, the protocol for allergen-specific immunotherapy has improved to increase efficacy and safety through coinjection or conjugation of allergens with an immunomodulatory adjuvant, premedication with an antihistamine or anti-human IgE antibody, or use of a rush protocol to shorten the duration of the updosing phase [4–8]. The injection route for allergens has also been examined in trials of modified allergens to shorten the schedule and to increase the safety for immunotherapy [9, 10]. In the last few decades, sublingual administration has been recognized as a route of administration of allergens that is safer than subcutaneous injection, and there is increasing evidence that the therapeutic effects of sublingual immunotherapy (SLIT) are comparable with those of traditional subcutaneous immunotherapy (SCIT) [11].

 In this review, we focus on the therapeutic effects of SLIT and the problems to be solved in future clinical studies. We also discuss recent findings for prospective and response-monitoring biomarkers for SLIT, and we examine the cellular mechanisms of SLIT.

## 2. Safety and Therapeutic Effects of SLIT

 Increasing numbers of clinical trials and meta-analyses have shown positive clinical effects and safety of SLIT. However, several case reports have also described anaphylactic shock or severe fatal reactions induced by sublingual administration of allergens [12–17]. In the reports, four patients experienced severe side effects with SCIT and discontinued the treatment prior to SLIT [15, 16]. Patients who have experienced severe side effects in SCIT may be at risk for a severe fatal reaction in SLIT. To prevent an allergen overdose, a tablet or solid form for sublingual administration may be better than the use of an atomizer or dispenser for administration of liquid allergens, especially for young children. Despite the few case reports of severe fatal events, life-threatening severe fatal reactions have not been found in clinical trials [18]. Therefore, SLIT is considered to be a safe treatment in which reactions such as anaphylaxis can be avoided by using correct clinical protocols.

 It takes a few weeks to six months to reach a maintenance dose using SCIT with a previous updosing phase to reduce the risk of side effects [19]. In some studies, a build-up phase is used for SLIT before administering the maintenance dose of allergens. A comparison of the clinical effects and safety among four different SLIT regimes for grass pollen allergy using a mixture of extracts of five grass pollens (*Anthoxanthum odoratum*, *Dactylis glomerata*, *Lolium parenne*, *Phleum pratense*, and *Poa pratensis*) concluded that a short build-up phase reduces the incidence of adverse events in administration of high-dose SLIT [20]. In this phase I study, the numbers of adverse events were compared among four active groups with a build-up phase repeating each concentration from 100 to 500 IR for 2 days (*N* = 6), a single daily build-up phase of 100 to 500 IR (*N* = 6), and no build-up phase for doses of 300 IR (*N* = 6) or 500 IR (*N* = 5). All groups showed mild and moderate adverse events, but only the group administered 500 IR without a build-up phase showed severe local adverse events (swelling of throat). A placebo group (*N* = 7) showed only mild adverse events. Another study compared the safety and efficacy among 3 SLIT groups with a build-up phase of 500 to 1,000 AU for 4 days, 300 to 1,200 AU for 4 days, and no build-up phase for a dose of 1,000 AU, using orosoluble tablets of a monomeric carbamylated allergoid [21]. Safety and efficacy were comparable among these groups, based on evaluation using a Visual Analog Scale (VAS), the Symptom Medication Score (SMS), and a nasal provocation test. An ultrarush schedule for SLIT has also been shown to be safe during the updosing phase, but severe systemic and local adverse events may occur in the maintenance phase [22, 23]. In contrast, urticaria has been reported to occur in an ultrarush protocol [24]. The safety of this protocol may depend on the type and biological function of the causal allergens. It has also been suggested that the build-up phase for SLIT can be omitted or shortened compared to that for SCIT [25].

 A recent meta-analysis found positive clinical effects of SLIT based on the results from 49 papers describing randomized, double-blind, and placebo-controlled (DBPC) trials [18]. The standardized mean differences (SMDs) for the symptom and medication scores were −0.49 (*P* < 0.00001) and −0.32 (*P* < 0.00001), respectively, in favor of active treatment (active; *N* = 2, 333, placebo; *N* = 2, 256). A meta-analysis of SLIT for grass pollinosis gave SMDs for the symptom (active; *N* = 1, 518, placebo; *N* = 1, 453) and medication (active; *N* = 1, 428, placebo; *N* = 1, 358) scores of −0.32 (*P* < 0.0001) and −0.33 (*P* < 0.0002), respectively, in favor of active treatment compared with placebo [26]. Both meta-analyses showed positive therapeutic effects of SLIT, especially for seasonal rhinitis, and these effects are comparable with those of SCIT [18, 27–29]. It has also been suggested that immunotherapy with SLIT and SCIT in combination may be beneficial [30]. In this study, 60 children with mild or moderate asthma or rhinitis who were monosensitized to house dust mite received injection of a mixture of *Dermatophagoides* allergens in a glycerinated solution. SCIT was used in a build-up phase for 16 weeks and was followed by SLIT three times a week as the maintenance phase. The clinical effects of SLIT were less than those of SCIT after 4 and 18 months and comparable after 12 months of treatment, based on the required dose of inhaled corticosteroids and the number of asthma attacks per year. SCIT and combination therapy of SLIT and SCIT significantly decreased the dose of inhaled corticosteroids and the number of asthma attacks at 4, 12, and 18 months and significantly improved the VAS for rhinitis. An advantage of SLIT is that sublingual self-administration can be performed at home during the maintenance phase, avoiding the need for patients to go to clinic for subcutaneous injection of allergens.

 There is also increasing evidence for clinical effects after an extended period of SLIT and for prolonged clinical effects after treatment [31]. SLIT in 24 children with respiratory symptoms due to monosensitization to house dust mite showed a lack of positive clinical effects in the first year, but significant amelioration of rhinitis and asthma in the second and third years compared to the first year of treatment [32]. A study of 137 patients allergic to house dust mite also showed clinical effects in 2-year and 3-year SLIT and prolonged therapeutic effects at 4 and 3 years, respectively, after these treatments [33]. Scores for nasal airway resistance, secretion, symptoms, and skin prick test were significantly reduced at the end of the first year, and the nasal secretion score was significantly reduced at the end of the second year of treatment. Two-year SLIT significantly attenuated nasal airway resistance, secretion, sneezing, symptoms, and skin prick scores at 1 and 4 years after treatment compared with the respective scores at the start of treatment although all scores except for nasal airway resistance at 4 years after treatment were slightly, but significantly, higher than those at the end of treatment. Three-year SLIT significantly attenuated these scores at the end of treatment, and total score of nasal airway resistance, secretion, and sneezing, score for the nasal airway resistance, and symptom score at 3 years after treatment were similar to or lower than those at the end of treatment.

**Figure 1:**
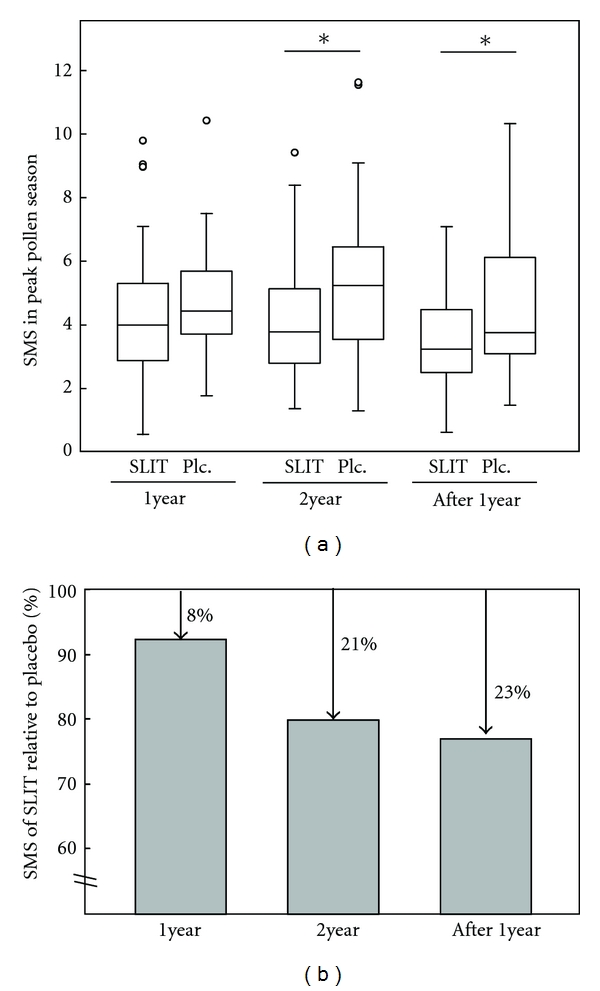
Clinical scores in 2-year SLIT and at 1 year after treatment [36]. (a) Average daily SMSs during a 2-year course of SLIT and at 1 year after treatment are plotted for the SLIT and placebo (Plc) groups. *P < 0.05 (unpaired Student *t*-test). (b) Percentage average SMSs for the SLIT group based on a value of 100% for the placebo group.

 Carry-over effects of SLIT are supported by other studies. DBPC trials of 3-year SLIT for grass pollen allergy showed significantly decreased scores for symptom and the rhinoconjunctivitis quality-of-life questionnaire (RQLQ), and SMS and the medication score tended to decrease with active treatment compared with those for placebo at 1 year after SLIT [34, 35]. Our recent results also suggest a 1-year prolongation of clinical effects after 2-year SLIT for Japanese cedar pollinosis [36]. Analysis of 88 participants (SLIT; *N* = 51, placebo; *N* = 37) showed positive therapeutic effects in the second year of SLIT compared with placebo (reduction of SMS by 21%, *P* = 0.02) and at 1 year after treatment (23%, *P* = 0.03) (Figure 1). A recent phase III trial performed as a large-scale randomized, DBPC study using a 75,000 SQ-T/2,800 BAU tablet in 257 subjects allergic to grass pollen also has shown that 3-year SLIT significantly decreased the mean rhinoconjunctivitis symptom and medication scores at 1 year after treatment compared with placebo. The results showed reductions of symptom scores of 31%, 36%, 29%, and 26% and reductions of medication scores of 38%, 45%, 40%, and 29% after 1, 2, and 3 years of treatment and after a follow-up year, respectively [37]. Long-lasting effects after 3-, 4-, and 5-year SLIT were evaluated in a 15-year prospective open controlled study in 59 patients with respiratory allergy for mite [38]. A decreased SMS of <50% of the baseline score (at the start of treatment) was found over the following 6 years after 3-year SLIT, and over 8 years after 4- and 5-year SLIT. The SMS after loss of the prolonged therapeutic effects increased to levels comparable with those in the control group. Significant clinical effects were obtained in a second course of SLIT given after the initial effects had vanished.

## 3. Unmet Problems in SLIT

 Compliance with self-administration at home may be an important factor in the therapeutic effect of SLIT. Compliance with SLIT is likely to be similar to that for other self-administered drug treatments for allergy [11], and education on the SLIT protocol is needed for good compliance [39, 40]. Checking the compliance of each patient based on the amount of remaining vials or tablets may also be important for evaluating the efficacy of SLIT in clinical trials [34]. A device that reminds patients about intake of allergens may be useful to achieve good compliance in long term administration and to improve the efficacy of SLIT [41]. Delivery as a tablet or solid form may be better than an aqueous solution using an atomizer or dispenser to achieve good compliance and to hold allergens stably under the tongue because human error or bad conditions of a nozzle may lead to administration of an inaccurate amount of liquid drops. Such mistakes may also increase the risk of adverse reactions [17].

 Bystander therapeutic effects of SLIT using allergens from a single source with polysensitized patients are uncertain. Inferior therapeutic effects for a polysensitized population have been reported compared with a monosensitized population [42]. Recent findings have shown that the use of both single and mixed allergen extracts improved mean QOL scores, increased threshold of a titrated nasal challenge, and decreased skin prick tests reactivity in polysensitized patients [43–45]. The efficacy of SLIT for polysensitized patients has also been found to be comparable with that for monosensitized patients [28]. Furthermore, SLIT for monosensitized (rhinitis only) and polysensitized (rhinitis and asthma) patients prevented or reduced additional sensitization compared with drug treatment [46, 47]. These preventive effects of SLIT were clearer for monosensitized patients. In contrast, SLIT for birch pollinosis was not effective against an already established apple allergy [48]. Mal d 1, a major allergen in apple, has 64% identity in amino acid sequence with Bet v 1, a major allergen in birch, and these allergens are cross-reactive in IgE-binding and T-cell activation. Bystander effects of SLIT using allergens from a single source for patients with other established allergies may depend on the allergens used for immunotherapy and the degree of sensitization to the allergy. Further clinical trials and meta-analyses are needed to evaluate the bystander and prophylactic effects of SLIT.

 In 2010, the World Allergy Organization defined a systemic reaction grading system for scoring of adverse reactions by SCIT to enable comparison of the severity of adverse events among clinical trials [49]. A similar approach to evaluation of clinical effects and adverse events in SLIT is needed to compare the clinical effects and therapeutic efficacy among studies that differ in allergen, dose, and method and protocol of administration. This will permit improved meta-analyses. Currently, it is difficult to optimize the SLIT protocol using results from multiple clinical trials that used different methods for evaluation of therapeutic effects, such as cumulative or average scores for symptoms and medication, QOL, VAS, local symptoms, and days with mild or severe symptoms over periods of days, months, seasons, and years [18, 50, 51]. It will be difficult to score the severity of allergic symptoms using the same grading system because both the pattern and main organ in which symptoms appear may differ among seasonal or perennial allergies in various areas. However, a scoring or grading system for use in scientific reports is needed as a minimum requirement to permit improved understanding by readers.

## 4. Trials of Adjuvant Therapy with SLIT

 Coadministration of an adjuvant with allergens may achieve more efficient and effective SLIT. Many studies in mouse models of asthma or rhinitis have shown increased effects of SLIT with adjuvant therapy. In most cases, the adjuvant is used to enhance development or activation of regulatory T cells (Treg) or increase adherence or permeability of allergens in sublingual mucosa to enhance uptake by antigen-presenting cells (APCs) such as mucosal dendritic cells (DC). Sublingual administration of an antigen conjugated with the nontoxic B subunit of cholera toxin to mice significantly induced antigen-specific Foxp3^+^CD4^+^ T cells in cervical lymph nodes and spleen and suppressed proliferation of cells from cervical lymph nodes after stimulation with antigen to a greater extent than that after treatment with the unmodified antigen. The serum TGF-*β* level was also higher after administration of the modified antigen compared to the unmodified antigen [52]. Sublingual coadministration of an antigen with either 1,25-dihydroxyvitamin D3 plus dexamethasone (VitD3/DEX) or* Lactobacillus plantarum* suppressed airway hyperresponsiveness (measured as PenH) compared with antigen alone, and coadministration with VitD3/DEX significantly induced Foxp3^+^ cells in mice [53]. Another mouse study supported the adjuvant activity of lactic acid bacteria in enhancing the therapeutic effects of SLIT [54]. A study using polymerized carbohydrate as a mucoadhesive adjuvant showed superior reduction of established airway hyperresponsiveness (PenH) and lung inflammation compared to administration of antigen alone or phosphate-buffered saline [55]. In this study, IL5 and IL10 production from splenocytes was reduced after stimulation with antigen in mice-administered antigen with adjuvant compared with mice-administered PBS or antigen alone. The therapeutic effects of adjuvant SLIT are also under evaluation in humans. In a Phase I/IIa study, coadministration of grass allergens with a high dose of monophosphoryl lipid A, an agonist for toll-like receptor 4, significantly increased the rate of negative findings in a nasal challenge test at two weeks after completion of 8-week treatment [56]. Further large scale studies are needed to evaluate the efficacy of adjuvant SLIT in humans.

## 5. Recent Findings on Biomarkers for SLIT

**Figure 2:**
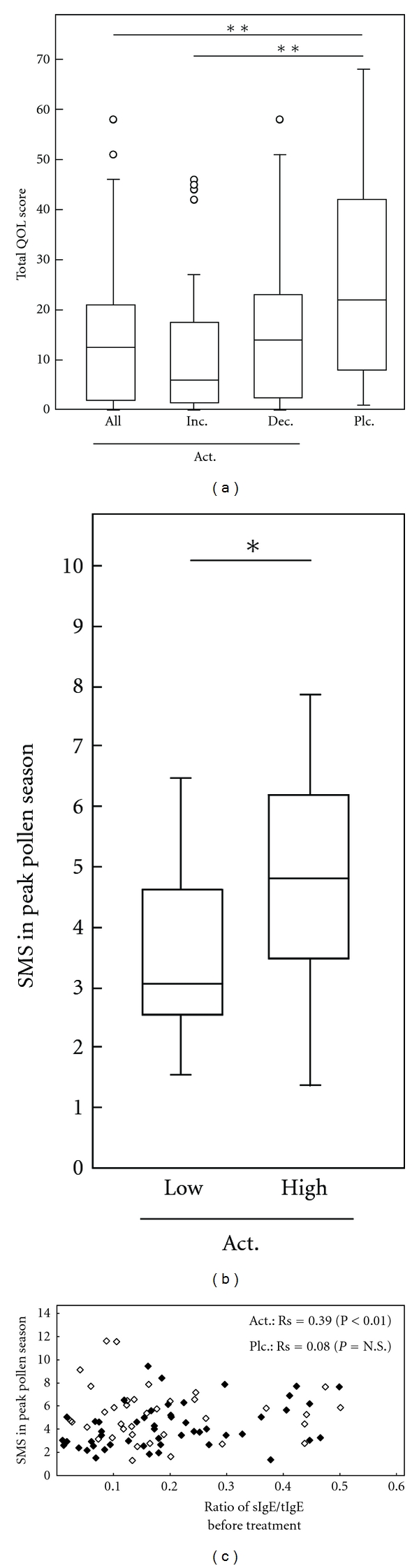
Response-monitoring and prognostic biomarkers for SLIT [36]. (a) Total scores from a QOL questionnaire are plotted for the SLIT group (All), a subgroup with increased antigen-specific Treg in the SLIT group (Inc), a subgroup with decreased antigen-specific Treg in the SLIT group (Dec), and the placebo (Plc) group after 2 years of treatment. **P < 0.01 (Mann-Whitney *U*-test). (b) SMSs in the peak pollen season for patients with low and high sIgE/tIgE ratios in the SLIT group (Act.). *P < 0.05. (c) Correlation between SMSs after 2 years of SLIT treatment and sIgE/tIgE ratios before treatment in the SLIT (Act, closed diamonds) and placebo (Plc, open diamonds) groups. Statistical data were obtained with Spearman correlation analysis. N.S.: not significant.

 Candidate biomarkers for response-monitoring or prognosis have been proposed and evaluated in many studies [4, 57, 58]. IL10 and Treg cells appear to be involved in the therapeutic mechanism of SLIT [59–61]. We reported upregulation of antigen-specific Treg cells (IL10^+^Foxp3^+^ cells) in CD25^+^CD4^+^ leukocytes from pre- to postpollen season as a response-monitoring biomarker for SLIT [36, 62]. Among patients treated with SLIT, total QOL and QOL-symptom scores after 2 years of treatment significantly improved in a subgroup with increased Treg cells compared with the placebo group, whereas the scores in a subgroup with decreased Treg cells were similar to those in the placebo group (Figure 2(a)). We also proposed that the ratio of antigen-specific IgE to total IgE (sIgE/tIgE) was a candidate as a prognostic biomarker for SLIT in a DBPC trial [36]. SMS in the SLIT group was correlated with the sIgE/tIgE ratio before treatment and was significantly improved in patients with a low sIgE/tIgE ratio compared to that in patients with a high sIgE/tIgE ratio (Figures 2(b) and 2(c)) [36]. The sIgE/tIgE ratio has been found to be significantly higher in responders than in nonresponders following 4-year immunotherapy [63]. In this study, responders to the immunotherapy (42 patients for SCIT and 103 patients for SLIT) showed higher grass- or mite-specific IgE/tIgE ratio than nonresponders (34 patients for SCIT and 100 patients for SLIT) evaluated with VAS score. In our trial, this ratio did not differ significantly between responders and nonresponders [36]. Further validation studies with a large sample size are needed before these biomarkers can be applied in the clinical management of SLIT.

 Upregulation of regulatory molecules after SLIT has been reported [57, 64] and programmed cell death ligand 1 (PDL1), IL10, and IgG4 may serve as response-monitoring biomarkers for SLIT [65]. In this report, all patients who received preseasonal, seasonal, and prolonged SLIT had increased percentages of PDL1^+^ and IL10^+^PDL1^+^ cells among CD14^+^ and CD19^+^ cells after stimulation with antigen in pollen season, compared to a placebo group. PDL1 is involved in induction and maintenance of Foxp3^+^CD4^+^ Treg cells in the presence of TGF*β* in mouse [66, 67], and induction of PDL1 may play an important role in induction of Treg cells by SLIT.

 Apolipoprotein is involved in lipid metabolism and lipid transport, and apolipoprotein E has roles in lipid antigen presentation and inhibition of T-cell activation [68, 69]. Upregulation of apolipoprotein A-IV (ApoA-IV) in serum in pollinosis patients from pre- to postpollen season was found to be significantly greater with SLIT than with placebo and was inversely correlated with SMS and QOL scores in the SLIT group [70]. ApoA-IV also significantly reduces histamine release *in vitro* from basophils taken from patients [70], and ApoA-IV induced by SLIT may be involved in downregulation of local or peripheral inflammation during the pollen season.

## 6. Mechanisms of SLIT

 Treg cells play an important role in suppression of Th2 responses and inflammatory cells [4, 71]. However, the cells that induce Treg cells after sublingual administration of allergens and the mechanism of induction remain unclear. DCs that preferentially induce Treg cells are thought to be located in the sublingual mucosa. In a mouse study, three types of DCs with different surface markers were identified within lingual and buccal tissue: CD207^+^ Langerhans cells in the mucosa, CD11b^+^CD11c^−^ and CD11b^+^CD11c^+^ myeloid DCs at the mucosal/submucosal interface, and B220^+^120G8^+^ plasmacytoid DCs [72]. Oral CD11b^+^CD11c^−^ DCs induced IFN-*γ* production by T cells, and oral CD11b^+^CD11c^+^ DCs and B220^+^120G8^+^ DCs induced IFN-*γ* and IL10 production by T cells in an antigen-specific manner. These oral DCs may preferentially skew development to antigen specific Th1 or Treg. The function of CD207^+^ Langerhans cells could not be determined because of limited cell numbers. In humans, oral mucosal Langerhans cells (oLCs) that constitutively express Fc*ε*RI on the surface have been found in atopic and nonatopic subjects [73]. Expression levels of Fc*ε*RI were found to be significantly correlated with serum IgE levels in atopic subjects. oLCs also expressed significantly higher amounts of major histocompatibility complex (MHC) I and II, CD40, CD80, and CD86 compared to skin Langerhans cells [73]. Toll-like receptor 4-ligation of oLCs has also been shown to induce production of IL10, TGF-*β*, IL2, IFN-*γ*, and Foxp3 [74], and oLCs might capture allergens within sublingual mucosa and present them to T cells to develop antigen-specific Treg cells [75, 76]. Further studies are needed to determine the importance of oral DCs and oLCs in the therapeutic mechanisms of SLIT.

 Induction of IgG and IgG4 as blocking antibodies in SLIT is still under debate [77, 78]. IgE enhances uptake and presentation of invading antigens by APCs via CD23, a process known as facilitated antigen presentation (FAP), and transcytosis by human airway epithelial cells in a CD23-dependent manner [79, 80]. The immune complex of IgE with antigen binds to CD23, and this binding leads to enhance antigen presentation by APCs to T cells [79]. There is increasing evidence to show that SLIT inhibits FAP by preventing binding of IgE with antigen or CD23 [81]. Decreased FAP after immunotherapy is correlated with T-cell activation *in vitro* and antigen-specific IgG titer, and FAP activity tends to correlate with IgE/IgG4 ratio and symptom score [82–84]. This inhibition of FAP leads to decrease antigen-specific proliferation and IL4, IL5, IL10, and IFN-*γ* production from T cells [85]. The inhibition persists over 2 years after discontinuation of 2-year immunotherapy although specific IgG and IgG4 levels decreased to preimmunotherapy levels [86]. Other factors may also be involved in the mechanism of FAP by inhibiting CD23 and IgE binding.

## 7. Conclusions

**Figure 3:**
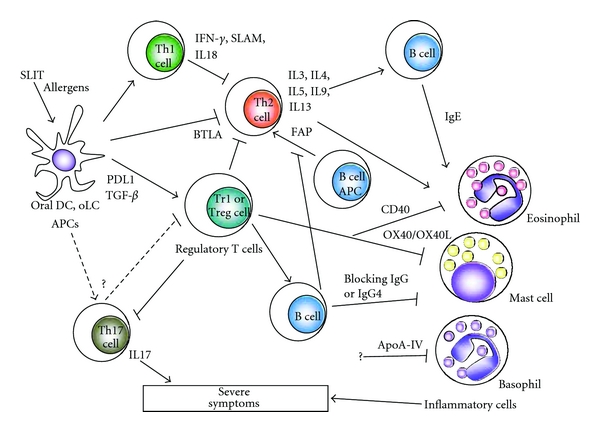
Proposed effects of SLIT on T cells, B cells, APC, and inflammatory cells [57]. Oral DCs or oLCs may take up allergens administered sublingually, followed by induction of Th1 or Treg cells to downregulate Th2 cells and inflammatory cells. The Treg cells also activate B cells to produce blocking antibody, which may inhibit binding between allergens and surface IgE on inflammatory cells to prevent secretion of inflammatory mediators and inhibit FAP by APCs or B cells to Th2 cells.

 One of the aims of immunotherapy is to induce tolerance against invading allergens. The therapeutic effects and efficacy of SLIT vary among allergies with different causal allergen sources. Achievement of a level of tolerance at which drugs are not required and symptoms are absent in the greatest numbers of patients requires further optimization of protocols and modification of SLIT or standardization of allergens as a SLIT vaccine. Adjuvant SLIT and combination with other methods may help to achieve more effective SLIT. The involvement of oral DC, oLCs, Treg, and FAP in the therapeutic mechanisms of SLIT has been proposed in many studies in humans and in mice (Figure 3). To determine the chain of mechanisms of SLIT, more studies are needed using human materials from clinical trials with large sample numbers. Understanding the precise mechanisms of SLIT should facilitate more effective immunotherapy for more patients with allergies.

## Abbreviations

APC: antigen-presenting cells
DBPC: double-blind, placebo-controlled
DC: dendritic cells
SCIT: subcutaneous immunotherapy
SLIT: sublingual immunotherapy
SMD: standardized mean difference
SMS: symptom-medication score
Treg: regulatory T cells
QOL: quality-of-life
VAS: Visual Analog Scale.
